# Predicting position along a looping immune response trajectory

**DOI:** 10.1371/journal.pone.0200147

**Published:** 2018-10-08

**Authors:** Poonam Rath, Jessica A. Allen, David S. Schneider

**Affiliations:** Department of Microbiology and Immunology, Stanford University, Stanford CA, United States of America; Universitat Pompeu Fabra, SPAIN

## Abstract

When we get sick, we want to be resilient and recover our original health. To measure resilience, we need to quantify a host's position along its disease trajectory. Here we present *Looper*, a computational method to analyze longitudinally gathered datasets and identify gene pairs that form looping trajectories when plotted in the space described by these phases. These loops enable us to track where patients lie on a typical trajectory back to health. We analyzed two publicly available, longitudinal human microarray datasets that describe self-resolving immune responses. *Looper* identified looping gene pairs expressed by human donor monocytes stimulated by immune elicitors, and in YF17D-vaccinated individuals. Using loops derived from training data, we found that we could predict the time of perturbation in withheld test samples with accuracies of 94% in the human monocyte data, and 65–83% within the same cohort and in two independent cohorts of YF17D vaccinated individuals. We suggest that *Looper* will be useful in building maps of resilient immune processes across organisms.

## Introduction

When we are infected, we suffer for a brief time, but then, ideally, we clear the infection and regain our health. This disturbance and recovery is known as “resilience” and depends upon a negative regulatory loop during which the host clears the infection and then returns to baseline. In a complex system such as the immune response, we observe sequential waves of activity where one variable is perturbed and then returns to baseline, to be followed by the next variable and so on. Together, these variables trace a multidimensional manifold through “disease space”. Though initially complicated, this manifold can be simplified by focusing on subsets of easily explained relationships. For example, we expect that some variables will have linear relationships with respect to each other if they are co-regulated. Likewise, when one variable induces a second, we might expect a sigmoid relationship, which can be useful because it is easy to describe mathematically and provides descriptive variables to measure vigor, slope, EC_50_ and maximum effect [1]. We find that variables that rise and fall out of phase with each other form loops in disease space. These loops are particularly useful because they can be used as maps to uniquely place an individual along the path of the infection [2]. Ultimately we would like to understand the properties that make a system resilient, to measure the strength of that resilience, and to eventually alter resilience within a single host. Our goal here was to develop a computational method to find variables out of phase with each other, forming loops.

Torres et al. [2] developed a systematic, albeit manual, approach to identify looping variables in large data sets. This approach was based on an assumption about the geometry of loops, reasoning that pairs of variables that enclosed large areas would be loops.

Here, we describe *Looper*, an automated computational method to identify pairs of variables in longitudinally sampled data that trace loops when plotted against each other. We test this approach on publicly available, longitudinal microarray datasets in humans under conditions of inflammation and resolution *in vitro*, in human monocytes (GSE47122 [3]), and, in response to vaccination with YF17D, in whole blood (GSE13699 [4], GSE13485 [5]). *Looper* identified candidate gene pairs for loops that capture the immune system dynamics in both the human monocyte cell culture as well as the YF17D vaccination data. We found that loops, traced when these variables are plotted against each other, provided a clear separation between the perturbation stages of inflammation and resolution. Perturbation encompasses the entire duration of inflammation and resolution following an insult, before the system has returned to near baseline levels. Therefore, time of perturbation corresponds to a stage along the inflammation-resolution spectrum. Using *Looper*, we found that we can predict the time of perturbation in withheld human monocyte samples with an accuracy of 94%. We experimentally validated this result in monocytes from independent human donors. Using the loops identified by *Looper* in the YF17D dataset, we achieved a perturbation stage prediction accuracy of 83% within the same cohort and 65–78% in two independent cohorts. We suggest that *Looper* would be useful in identifying loops under diverse health-perturbing conditions across organisms, driving the prediction of infection stages using these maps.

## Results

### Using loops to predict position on the inflammation/resolution trajectory in human monocytes

All of our analyses were performed on publicly available gene expression datasets. We downloaded the datasets from the National Center for Biotechnology Information Gene Expression Omnibus (GEO; accession numbers GSE47122, GSE13699, GSE13485) using the *Metageo* Python module.

**Fig 1:**
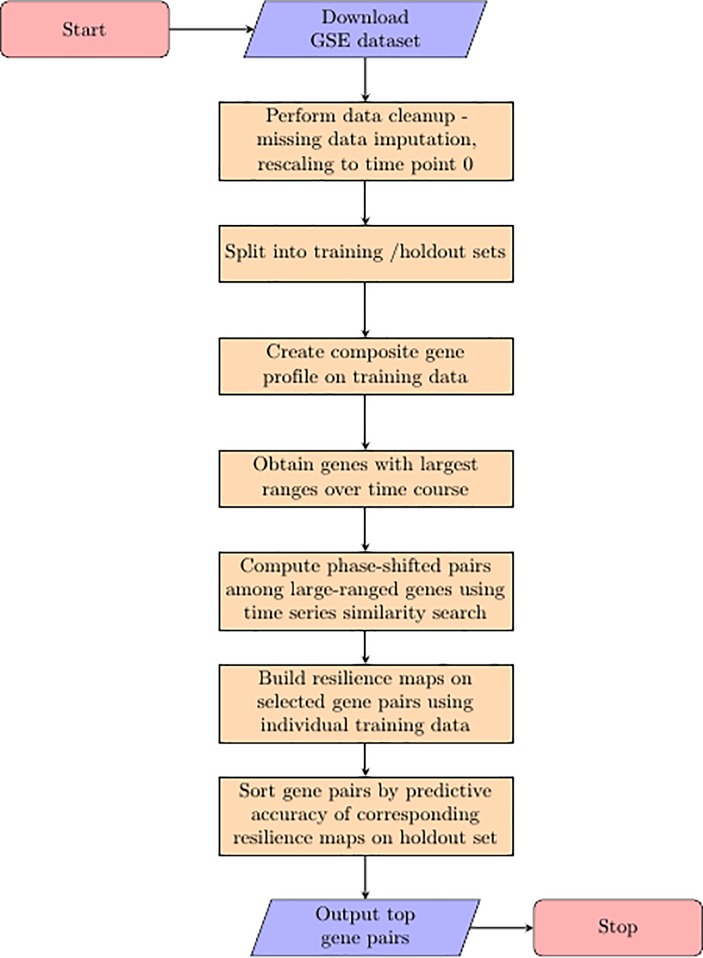
Workflow of loop identification GSE raw datasets were processed using *Metageo* to obtain gene expression data with corresponding gene names.

We sought to identify gene pairs that traced looping paths through a two dimensional phase space and started by using microarray data gathered from human monocytes sampled over the course of inflammation and resolution in an *in vitro* model of inflammation (GSE47122 [3]) **(Fig 1).** We chose this dataset because it captured a self-resolving immune response sampled long and densely enough to map the entire course of inflammation and resolution. In this study, monocytes extracted from 12 human volunteers were sequentially treated *in vitro* with CCL2, LPS, TNF*α* and IFN*γ* as inflammatory stimuli followed by IL10 and TGF*β* as macrophage deactivating stimuli. These treatments were meant to mimic the inflammation and resolution experienced by macrophages during an infection in vivo. Cells were sampled for microarray analysis for a total of 9 time points at 0, 2, 2.5, 3, 3.5, 4, 14, 24 and 48 hours post culture, and gene expression was scaled to log2. In the original study, among the 12 donors, 3 were sampled at 0–3.5 hours while the remaining 9 donors were sampled at 0 and 4–48 hours. Accordingly, instead of splitting the GSE47122 dataset into training and test sets based on individuals, we split it such that the training set comprised approximately of 50% of the data points sampled randomly but evenly to cover all time points. We then applied *Looper* to identify looping gene pairs within the training set as follows.

Using the training data, *Looper* obtained the median gene expression for each time point, creating a composite profile that we centered to time point 0. From this profile, *Looper* filtered for those genes whose expression ranges were in the top 0.5% of all genes under analysis. We set 0.5% as an arbitrary cutoff to generate a subset of genes with a dynamic expression range that would allow us to detect phase-shifts in the following steps. Using these filtered genes, *Looper* isolated those gene pairs that were phase-shifted using the symbolic aggregate approximation (SAX) algorithm described in the methods. Specifically, each gene expression time series was converted into a symbolic string representation using SAX. This conversion was performed by the SAX algorithm as follows: each gene expression vector, comprised of gene expression values across all time points, was normalized to a standard normal distribution, and these normalized amplitudes were then assigned symbols based on the regions of a standard normal distribution into which they fell.

**Fig 2:**
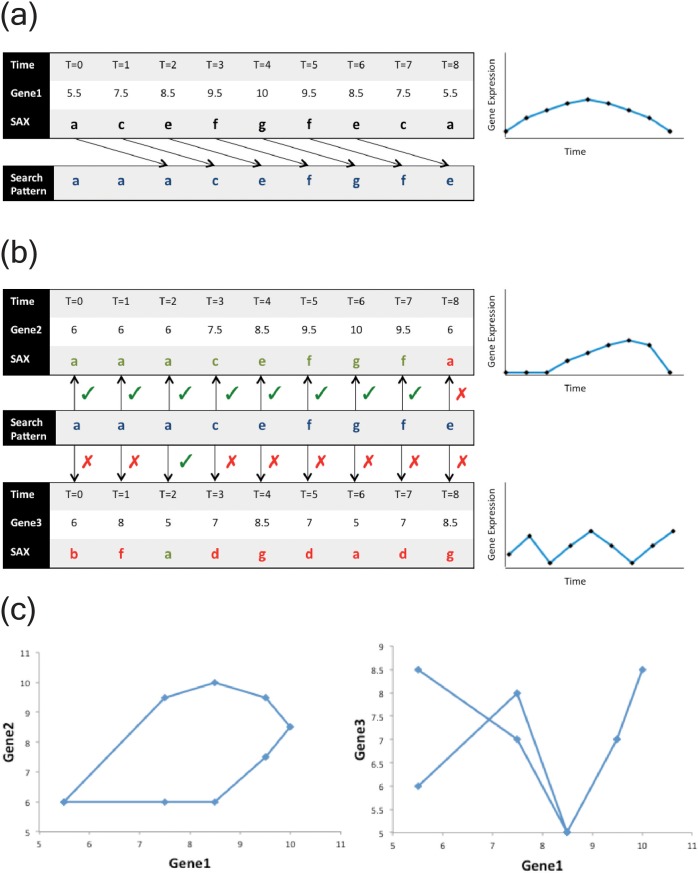
Schematic demonstrating use of symbolic aggregate approximation (SAX) in identifying candidate phase-shifted gene pairs. (A) Selected large-ranged genes were converted from numerical log2 form to SAX form. For each gene, we then computed a search pattern by shifting the SAX form by T/4 time points (T = total number of time points sampled) (B) Determining phase-shifted gene pairs based on SAX search pattern match; Genes 1 and 2 match the pattern while Genes 1 and 3 do not. (C) Genes that match the search pattern trace a loop (left) while mismatched genes do not (right).

We used a search pattern equivalent to a 90 degrees phase-shift in the gene expression; when two vectors are shifted by 90 degrees with respect to each other, they trace a perfect circle. The phase-shift by 90 degrees corresponded to a shift in the original pattern by T/4, where T is the total number of sampled intervals in the data. In this case, since the data was sampled 9 times, each SAX pattern was shifted by 9/4, i.e. 2 symbols to create a corresponding search pattern **(Fig 2).**

**Fig 3:**
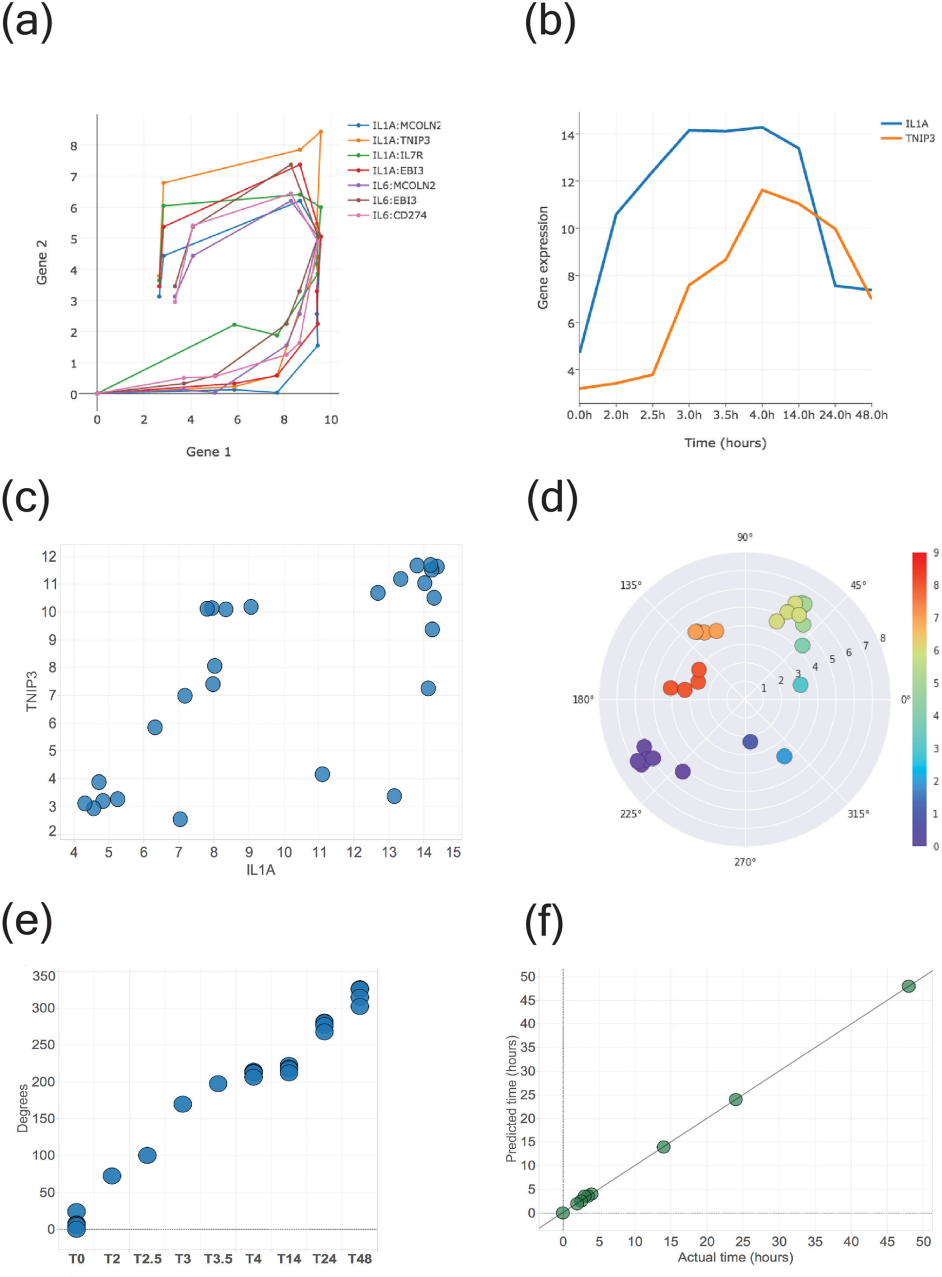
IL1A-TNIP3 loop in human monocytes can predict perturbation stage in test samples. (A) Gene pairs tracing loops identified using training data from the human monocyte dataset (GSE47122). Each loop represents the space traced by a gene pair using median gene expression across individuals. (B) Gene expression profile of IL1A and TNIP3 highlighting phase shift. (C) IL1A-TNIP3 loop. Circles represent data points for all individuals and time points in the training data. (D) Polar plot derived from IL1A-TNIP3 loop. Points are colored based on an ordinal time scale to clearly distinguish between points sampled at different times (0 on this ordinal scale corresponds to time point 0 hrs, 1 to 2 hrs, 2 to 2.5 hrs, 3 to 3 hrs, 4 to 3.5 hrs, 5 to 4 hrs, 6 to 14 hrs, 7 to 24 hrs, and 8 to 48 hrs). Distinct time points can be seen to occupy distinct regions on the plot. (E) Angle, derived from polar transformation of the loop, is positively linearly correlated with time on the x-axis (Pearson’s *ρ* = 0.98) (F) Prediction of perturbation stage for 34 test samples, computed using k-nearest neighbors, shows an accuracy of 94% with a strong linear relationship between predicted and actual time (*R*^2^ = 0.99).

Once SAX representations and search patterns for each filtered gene had been computed, *Looper* identified those gene pairs whose SAX representation most closely matched some other gene’s search pattern. Using this method, *Looper* generated an exhaustive list of gene pair candidates that are phase-shifted in such a manner as to trace a loop in the training data **(Fig 3A).** Among other gene pairs, the time course of gene expression showed that the inflammatory cytokines IL1A and TNIP3 (TNFAIP3-interacting protein) are phase-shifted in their expression **(Fig 3B, S1 Fig).** IL1A and TNIP3 have been suggested to be involved in a feedback mechanism, where IL1A expression precedes that of TNIP3, which in turn suppresses NF-kB driven induction of IL1A [6]. Since IL1A and TNIP3 traced a clear loop, we decided to further investigate this gene pair **(Fig 3C).** To determine whether the trajectory recapitulates time in the IL1A-TNIP3 space, we transformed the data from Cartesian to polar coordinates and plotted the resulting angles of the samples versus time **(Fig 3D).** The angle derived from polar transformation showed a positive linear correlation with time (Pearson’s *ρ* = 0.98) **(Fig 3E).** Similar to the training data, the angles derived from all data points also showed a positive correlation to time **(S2 Fig).**

We sought to determine whether we could predict time of perturbation of withheld samples using the IL1A-TNIP3 loop generated with the training data. We performed K-nearest neighbor analysis (K = 3), determined the nearest neighbors of each withheld data point in the IL1A-TNIP3 two-dimensional space, and assigned to it a time point that corresponded to that of the majority of its neighboring training samples. This approach predicted the time of perturbation in withheld samples with an accuracy of 94% **(Fig 3F).**

We performed similar analyses with 9 other gene pairs that traced loops, similar to that traced by IL1A-TNIP3 and obtained prediction accuracies **(S1 Table).** Among these looping gene pairs, IL1A-TNIP3 yielded the highest prediction accuracy. Randomly sampled gene pairs yielded poor prediction accuracies, and did not fall into the same distribution as the accuracies obtained from looping gene pairs identified by *Looper* **(S3 Fig).**

### Experimental validation of IL1A-TNIP3 loop in human monocytes

**Fig 4:**
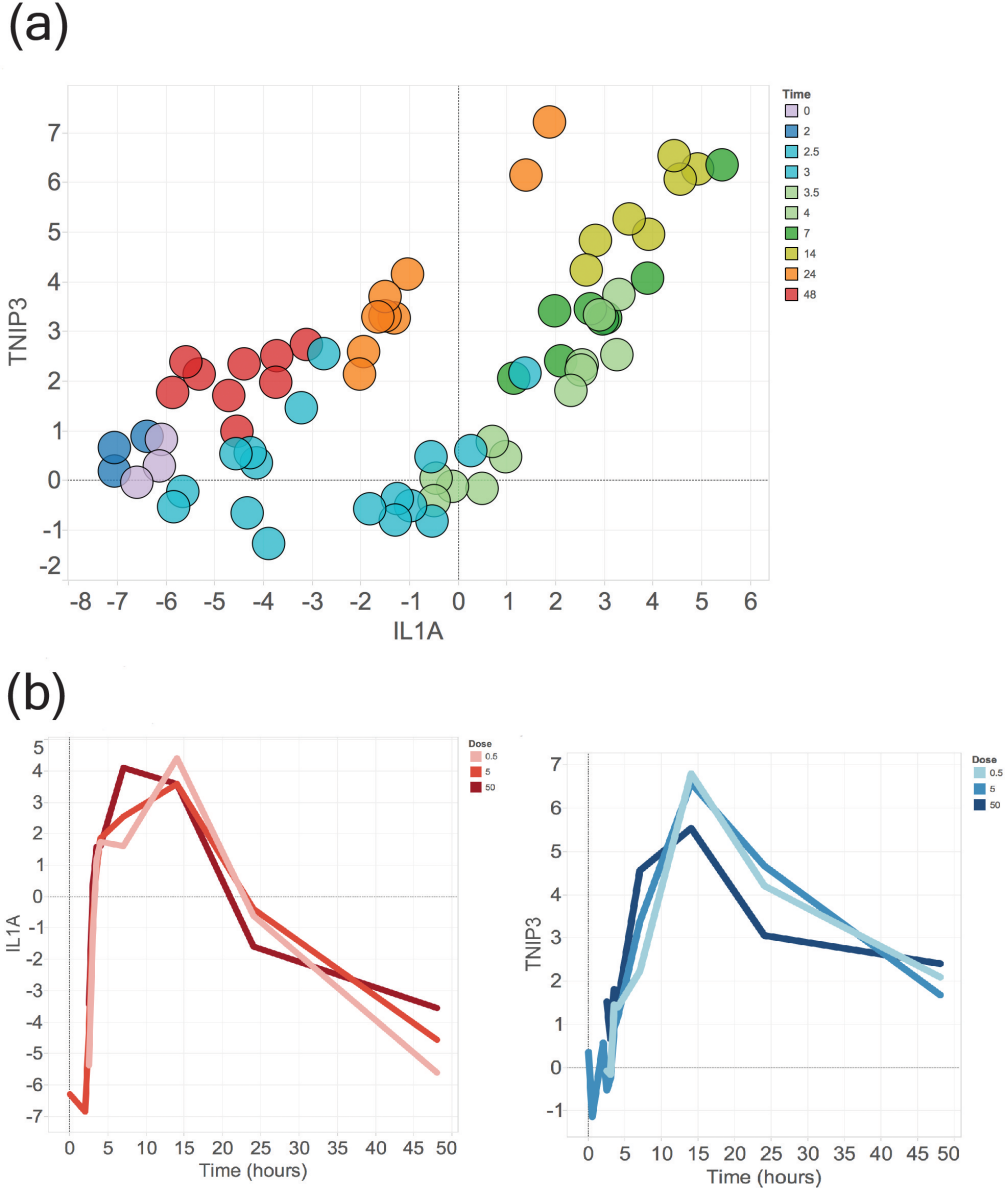
Experimental validation of IL1A-TNIP3 loop. (A) Experimental confirmation of IL1A-TNIP3 loop in a representative human donor among a total of three donors. Circles represent technical and biological replicates. Colors represent time points, labeled next to the figure; distinct colors represent distinct exposures to given stimuli described in the Results. Different doses of LPS were used (5ng/ml, 50ng/ml and 500ng/ml) but a dose-dependent response was not observed. (B) Gene expression of IL1A (red, left) and TNIP3 (blue, right) over time highlighting phase-shift. Increasing color intensities correlate with increasing doses of LPS.

To validate the IL1A-TNIP3 loop experimentally, we repeated the experiment described in the GSE47122 study using monocytes purified from whole blood from 3 independent donors. We cultured monocytes and gathered samples longitudinally to assess gene expression using qRT-PCR. A phase-shift in the IL1A-TNIP3 expression profiles was confirmed across all donors and when plotted against each other, the gene pair traced a loop, supporting our data mining-driven discovery using *Looper* **(Fig 4, S4 Fig).**

### Independent validation of prediction accuracy of loops identified by *Looper* in YF17D-vaccinated individuals

Our next goal was to extend the use of *Looper* to identify loops from longitudinal immune data gathered *in vivo*, and validate the power of perturbation stage prediction of the resulting looping maps in independent datasets. We chose a multi-cohort longitudinal gene expression dataset gathered from whole blood samples of humans following vaccination with Yellow Fever Vaccine 17D (YF17D) (GSE13699 [4]). This dataset fulfilled two criteria: first, there were 7 time points sampled between days 0 and 60 in one of the cohorts (Montreal), more than our cutoff of 6 data points. Second, this study included another cohort (Lausanne) of 13 individuals sampled at days 0, 3 and 7 post-vaccination (39 data points), which rendered it suitable for use as an out-of-sample dataset to test the accuracy of our loop in predicting the position along the inflammation-resolution trajectory. An additional independent YF17D vaccination dataset (GSE13485 [5]), which is comprised of 10 individuals sampled longitudinally at days 0, 3 and 7 post-vaccination (the Emory cohort), ensured that we could further validate the use of the looping map generated from the Montreal cohort in predicting perturbation stages.

To assess the overall structure of the Montreal cohort, we performed topological data analysis (TDA) on a subset of 91 genes, comprising the top 0.5% of the total number of genes sorted by standard deviation with respect to baseline at day 0. Because a topological network does not impose any shape on the data, unlike other methods such as hierarchical clustering or dendrograms, the natural shape of the data is preserved [7, 8] [9]. TDA in high dimensional space on the subset of genes revealed that the gene expressions loop back to return to baseline levels (day 0). However, most changes in gene expression returned to baseline levels by day 14 while days 3, 7 and 10 occupied distinct areas on the network **(S5 Fig).** Our observation is consistent with the results from Principal Components Analysis in the original study. Therefore, we dropped day 60 from our analyses, and focused on predicting days 3, 7 and 10 as ‘early’, ‘middle’ and ‘late’ stages of perturbation in the test data set, respectively. In addition, days 0, 14 and 28 were treated as interchangeable when predicting time post-vaccination (perturbation stage), mapping to a ‘base’ stage.

**Fig 5:**
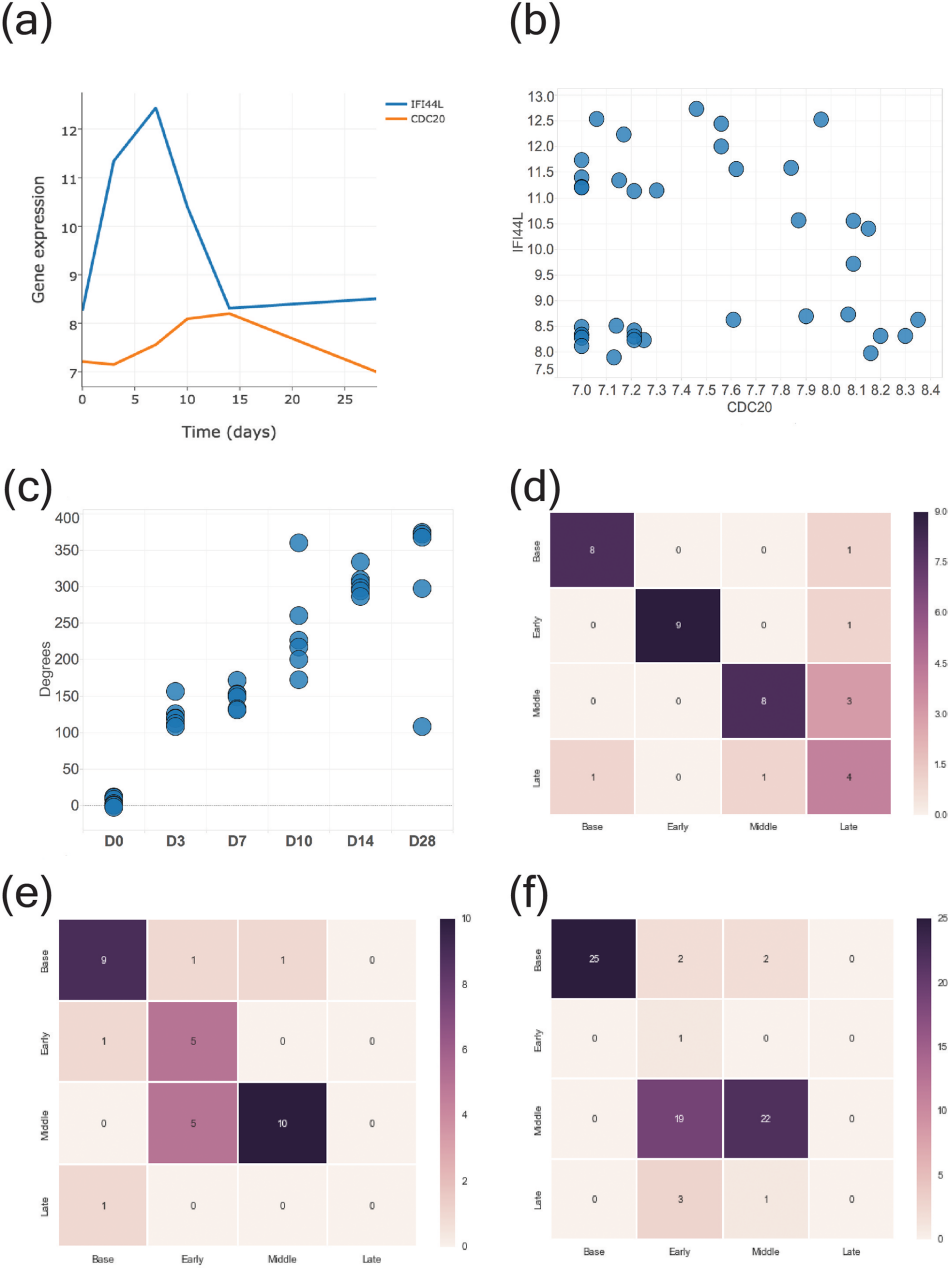
Identifying CDC20-IFI44L loop and predicting perturbation stage in YF17D-vaccinated individuals. (A) Timeline of gene expression shows that CDC20 peaks at day 3 whereas IFI44L peaks at day 7 for training data in the Montreal cohort (n = 11) (GSE13699). (B) CDC20-IFI44L loop. Circles represent data points for all individuals and time points in the point cloud. Two data points have been removed for better visualization; all data points are shown in S6 Fig. (C) Angle derived from polar transformation of the CDC20-IFI44L loop is positively linearly correlated with and time (Pearson’s *ρ* = 0.91) (D) Confusion matrix, in the form of a heatmap, showing prediction accuracy for stage of infection in the withheld samples in the Montreal cohort (n = 7) (GSE13699). A stage prediction accuracy of 83% was achieved. (E) Confusion matrix, in the form of a heatmap, showing prediction accuracy for stage of infection in the Lausanne cohort (n = 11) (GSE13699). A stage prediction accuracy of 73% was achieved. (F) Confusion matrix, in the form of a heatmap, showing prediction accuracy for stage of infection in the Emory cohort (n = 25) (GSE13485); a prediction accuracy of 65% was achieved.

Our goal was to use looping gene pairs identified from the Montreal cohort to predict perturbation stage in the Lausanne and Emory cohorts. To this end, we split the Montreal cohort comprised of 15 individuals such that 11 individuals were used as training data and 4 individuals as test data. As opposed to the human monocyte data, we did not generate a composite gene expression profile with the training samples. Instead, for each individual in the training data, using *Looper*, we identified gene pair candidates that traced a loop, finally selecting those gene pairs that were common across all individuals in the training data. CDC20-IFI44L was one such gene pair; the gene expression profiles demonstrated a phase-shift, with IFI44L (Interferon- induced protein 44-like) peaking before CDC20 (Cell division cycle 20) **(Fig 5A).** Because CDC20-IFI44L traced a clear loop, we decided to follow up on this pair **(Fig 5B, S6 Fig).** The data samples were transformed to obtain polar coordinates and centered; the angles derived from this loop were found to correlate linearly with time (Pearson’s *ρ* = 0.91) **(Fig 5C).** Using K-nearest neighbor analysis (K = 3) on the CDC20-IFI44L loop generated from the Montreal cohort, we achieved an accuracy of 83% in predicting the perturbation stage in withheld test samples, comprised of 4 individuals (24 data points) in the Montreal cohort **(Fig 5D).**

Leave-one-individual-out-cross-validation (LOOCV) analysis on all 15 individuals in the Montreal cohort resulted in a tight range of prediction accuracy, implying that there were no outliers in the data **(S2 Table).** Next, using the same CDC20-IFI44L loop derived from the training data in the Montreal cohort, for the 33 data points in the Lausanne cohort sampled at days 0, 3 and 7, the perturbation stage was predicted with an accuracy of 73% **(Fig 5E).** Days 0, 3 and 7 are referred to as base, early, and middle stages, respectively. Finally, we validated the predictive power of the CDC20-IFI44L loop in identifying perturbation stage in another YF17D vaccination study GSE13485 (Emory cohort), regarded as an independent validation set. The Emory cohort consists of PBMCs sampled from a total of 25 individuals in two separate trials, at days 0, 3 and 7 post-vaccination. Using the CDC20-IFI44L data from the Montreal cohort, the perturbation stage for the 75 data points in the Emory cohort was predicted with an accuracy of 65% **(Fig 5F).** Next we performed *in silico* sensitivity analysis to measure the robustness of our method in predicting perturbation stage for noisy out-of-sample data. The addition of noise, sampled from a Gaussian distribution centered on the mean expression profiles of the CDC20-IFI44L gene pair, along with its corresponding standard deviation, to the Emory cohort resulted in a shift in prediction accuracy from 65% to 63%, suggesting that our proposed method can handle noise.

## Discussion

Ideally, when infected, our bodies enter a state of sickness but regain health thereby demonstrating resilience. The dynamicity of the immune response to an infection is reflected in the gene expression profile of an individual; mapped across the full spectrum of perturbation that includes inflammation and recovery, this expression profile can represent the overall resilience of the individual. Studying resilience allows us to move beyond the binary identification of the presence or absence of infection and investigate the reasons why some individuals recover poorly or not at all.

We study resilience by focusing on looping data, and we define loops as paths traced by two out of phase variables in phase space. We describe *Looper*, a parsimonious computational approach to identifying looping gene pairs, and we demonstrate this approach through the analysis of publicly available, longitudinally gathered microarray datasets in humans under conditions of inflammation and resolution in vitro, in human monocytes, and in response to vaccination with YF-17D, in PBMCs. The main constraint we used to select datasets for our analysis is that the data should represent inflammation as well as resolution, so that a complete trajectory from infection to recovery can be mapped. As long as the time points at which the data have been gathered show dynamic gene expression profiles, with gene expression levels rising and then returning to near-baseline levels, we can use the data to detect loops. Even though the publicly available human monocyte dataset (GSE 47122) was unevenly sampled across time, we were able to detect the dynamic expression profiles of genes. We assume here that the rationale behind such uneven sampling is informed by the researchers’ prior knowledge of gene induction kinetics of inflammation and resolution in this specific model.

For each of these datasets, *Looper* identified candidate gene pairs tracing loops that capture their respective immune system perturbation dynamics. We suggest that these loops can serve as maps by providing a clear separation between stages of inflammation and resolution, and we provide evidence in support of our hypothesis by using such maps to predict the position along an inflammation/resolution trajectory in test samples in both datasets with considerable accuracies.

Genes that are induced and then resolved over an infection are often functionally coupled but have varied temporal relationships. Two genes can be expressed concurrently, at mutually exclusive points in time, or in a phase-shifted manner where the induction of one gene precedes that of the other but shares a partial overlap. In our workflow, we focus on identifying these phase-shifted gene pairs. Phase-shifts are not unique to the datasets we have studied; they can be found in infections such as malaria (in mice and in humans), not just in gene expression data but also between two behavioral variables such as temperature and weight [2]. Further, gene expression data in oscillating systems such as the cell cycle [10] and circadian rhythm [11] appear to have functionally coupled phase-shifts, though these studies do not analyze gene expression data in phase space. Arner et al. [12] show that during cellular differentiation, genes encoding enhancers that modulate transcription factors undergo coordinated phase-shifts.

The gene pairs we identify in our studies are predicated upon the choice of phase shift derived from the number of time points in the corresponding data. As more longitudinal data is gathered in the field to cover entire trajectories of resilient individuals, we expect to identify new gene pairs. However, the gene pairs we identify in our studies have been suggested to be functionally linked [6][13]. In the IL1A-TNIP3 map of human monocyte behavior, IL1A induction precedes that of TNIP3. This can be explained because TNIP3 (TNFAIP3-interacting protein), the interacting partner of TNFAIP3 (TNF-alpha-inducible protein 3), though induced by LPS, is a negative regulator of NFk-B signaling which induces IL1A transcription [6]. Given that the expression of TNIP3 facilitates inhibition of NF-kB activation, the observed phase-shift appears to represent a feedback mechanism. A functional link between CDC20 (Cell division cycle 20) and IFI44L (interferon-inducible 44 like), the gene pair identified by *Looper* from the YF17D vaccinated individuals [4], has not been suggested previously. IFI44L is a paralog of IFI44, a well-known immune response gene induced in response to viral infections [14]. CDC20, reported to be upregulated in the original study (GSE13699), is involved in cell division and might be an indicator of proliferating immune cells in the PBMC population during the vaccine induced immune response. CDC20 is known to be upregulated in NK cells undergoing proliferation in PBMCs during malaria infection in mice [15].

The use of loops as maps to predict the stage of perturbation has potential value in clinical settings. Patients presenting with an infection are likely to occupy different areas of the map that correspond to their specific stage of infection or perturbation. Unlike controlled laboratory experiments, we have no knowledge of the precise time of start of their infection, and typically we have access to a single, cross-sectional datapoint. This problem has been explored in the context of *Arabidopsis thaliana* infection to develop methods of determining pseudo-time as a proxy for real time [16]. Identifying the perturbation stage in single samples using our loops as maps may allow for personalized medical intervention by distinguishing patients on a path to morbidity from ones that are safely past their nadir and on a clear path to recovery. There can be other paths traced between the extremes of morbidity and full recovery. For instance, in an individual that has cleared infection but is far from full recovery because of its poor biological repair mechanisms, alleviating damage might be a priority [1][17], and identifying such individuals or their stage of infection is critical. By contrast, identifying individuals that are on a path to naturally clearing their infections could reduce our use of antibiotics. While some studies on infections such as sepsis have come up with gene signatures to distinguish between the presence or absence of infection along with its severity [18], our analysis instead uses gene pair measurements covering the stages of infection and recovery, thus allowing us to trace the resilience of the system and predict the perturbation stage for new individuals.

We observed a range of predictive accuracies across the datasets analyzed. The highest accuracy for perturbation stage prediction was achieved at 83% for the withheld samples within the Montreal cohort, which was used to identify the loops. The accuracy of perturbation stage prediction was 65% for samples in the Emory cohort (GSE13845) as compared to 73% for the Lausanne cohort dataset (GSE13699); this discrepancy could be due to differences in the platforms used in both experiments, differences across humans, or differences in the actual rate at which individuals pass through the loop. Our intent here was not to discover biomarkers for use in a clinical setting; instead we sought to develop a simple approach to automate the discovery of loops that summarize resilience in diverse datasets. Once we establish that a gene pair robustly generates loops across different individuals, we intend to study the gene pairs and their molecular pathway in-depth, for example by examining how disrupting the expression of one gene or its feedback loop impacts the induction kinetics of the other gene in the gene pair.

In our studies we have demonstrated that these looping gene expression patterns can be found in immune cells such as monocytes as well as in PBMCs. It will be interesting to see if there are common loops across different cell types and across a diverse panel of immune system perturbations. Deviations from a healthy loop could reveal biological insights; for instance, in a typically self-resolving malaria infection, recovering mice trace a small loop while dying mice diverge and trace larger arcs [2]. As the field gathers more longitudinally sampled datasets under different health-perturbing conditions and in diverse cohorts, we can begin to identify robust loops that summarize resilience and serve as maps. Healthy individuals sampled over six months show little change in gene expression as measured by microarray, suggesting that robust baselines can be obtained [19]. With adequately rich datasets, we can better understand the dynamics of the gene pairs constituting the maps, identify infection stages with greater precision and discover novel ways to nudge patients back to health.

## Methods

### Data selection, collection and processing

Publicly available human gene expression (microarray) datasets were selected with an eye to experiments that sampled patients longitudinally from the start of the perturbation through to resolution of inflammation, and included at least six time points. Our rationale for this prerequisite was that we needed variation in two variables that rose and fell out of phase with each other, which requires a minimum of four samples. Automated search could not be effectively applied at this step. Though several datasets have multiple data point samples, they focus on a narrow stage of the infection and don’t include recovery. We chose microarray datasets following an extensive literature search using the key phrases “*longitudinal*”, “*microarray*”, “*inflammation*”, and “*resolution*”. Selected gene expression datasets were downloaded from the National Center for Biotechnology Information Gene Expression Omnibus (GEO; accession numbers GSE47122, GSE13699, GSE13485), using a Python module *Metageo*, that we designed and uploaded to the Gitlab repository https://gitlab.com/prath/resilience2018. *Metageo* extracts the GEO Platform Files (GPL) and maps the probe IDs on the microarray file to gene names on the GPL file, generating an output file with gene names replacing their corresponding probe IDs. For multiple probe IDs that map to the same gene, *Metageo* computes the median gene expression and assigns it to the gene.

### Nearest neighbor analysis

K-Nearest Neighbor analysis was performed in Python using the provided code (https://gitlab.com/prath/resilience2018). Nearest neighbor computation was performed with K = 3 using Euclidean distance as the metric to measure similarity.

### Polar transformation and statistical analysis

Data was transformed from Cartesian to polar coordinates using MATLAB code described previously [2] (https://github.com/bytorres/PlosBio2015). Briefly, this script finds the center of a two-dimensional point cloud by first normalizing the ranges of the two dimensions. Multiple possible centers are tested within the range of the data points and the center is identified as one that produces the smallest variance for calculated radii. We defined 0 degrees as day 0 of experiment. Transformed data were centered and visualized and graphed using Tableau v9.0.

### Topological data analysis

Topological data analysis (TDA) was performed on the YF17D-vaccinated Montreal cohort (GSE13699) with the Ayasdi 3.0 software platform (Ayasdi Inc., Menlo Park, California). Nodes in the network represent clusters of human samples, and edges connect nodes that contain samples in common in terms of their gene expression profiles. Details of the TDA workflow are described in Torres et al [2]. The following variables were used: Gain: 5, resolution: 23, metric: Normalized correlation, lenses: MDS1 and MDS2.

### Symbolic aggregate approximation (SAX)

We used Symbolic Aggregate Approximation (SAX) [20] to convert the time course of gene expression data into string sequences and perform a pattern search for phase-shift behavior. SAX is a time-series similarity search algorithm that computes string representations of time series via vector quantization and piecewise aggregate approximation. Piecewise approximation, in a close analogue to sampling, enables the technique to reduce the search space to an amenable number of dimensions, while vector quantization enables the preservation of magnitude and range information in the signal. Finally, representing the aggregated and quantized signal as a string enables users to leverage optimized string-matching and pattern-matching algorithms that are honed for speed. Overall, due to its symbolic nature, the SAX algorithm lends itself well to performing time-series similarity search across high-dimensional data spaces.

A related methodology of plotting infdividual trajectories through multidimensional disease space described previously [21] represents longitudinal trajectory in two dimensions in the form of a symbolic sequence where each symbol represents a change in direction of the vector between two consecutive time points. Our analysis differs by representing each gene expression profile as a single string sequence using SAX, and defining candidate gene pairs based on overall phase-shifted similarities between their two corresponding SAX patterns.

### *Looper*: Identifying loops using gene expression data

We packaged our methodology of identifying loops using gene expression data in the form of a computational module written in Python; we have named this module *Looper* since it discovers loops in disease space, and have made it available in a GitLab repository: https://gitlab.com/prath/resilience2018. A schematic of the *Looper* workflow is described in Fig 1, and an explanation of the methodology follows.

Each selected dataset described gene expression values sampled across multiple time points for a collection of individuals in response to inflammation or vaccination. For each dataset, we imputed missing time points in individuals using the median gene expression for that time point across all other individuals. The gene expression data was then rescaled to baseline (time 0), and split into training and holdout sets. Next, a composite gene expression profile was created by taking the median of gene expression data per time point across all individuals within the training data. To capture dynamic transcripts, we filtered the data down to a list of top 0.5% genes that showed the largest expression ranges across time as measured by standard deviation. Through this filtering step, we recovered 95 genes out of an initial 18859 genes in the in vitro human monocyte dataset (GSE47122) and 91 genes out of an initial 18197 genes in the YF17D vaccination dataset (GSE13699). At this point, there were 4465 and 4095 possible gene pairs in the in vitro human monocyte dataset and the YF17D dataset, respectively. To identify the subset of gene pairs that were phase-shifted with respect to each other, we performed a time-series similarity search using symbolic aggregate approximation analysis (SAX), described in the methods, on the filtered list of genes. We ultimately obtained 102 and 45 phase-shifted gene pairs in the in vitro human monocyte dataset and the YF17D dataset, respectively. The phase-shifted gene pairs were plotted against each other to visually confirm loops. The gene pairs that traced loops were used to predict time post perturbation in the holdout sets, and sorted based on their prediction accuracies.

### Human monocyte cell culture

Experimental validation was performed following the protocol described by Italiani et al. [3] used to obtain the human monocyte dataset GSE47122. Whole blood was procured from healthy donors from the Stanford Blood Center (Stanford, CA). Briefly, monocytes were purified from PBMCs, isolated from whole blood by Ficoll-Paque PLUS (GE Healthcare) gradient separation using CD14 MicroBeads (Miltenyi Biotec), counted and plated at a density of 100,000 cells per well in 96 well plates in 200*μ*l RMPI-1640 (GIBCO, Life Technologies, Paisley, UK) supplemented with 50*μ*g/ml gentamycin (GIBCO) and 5% heat-inactivated human serum. Monocytes were sequentially exposed to CCL2 (10 ng/ml), TNF*α* (10ng/ml), LPS from E. coli serotype 055:B5 (5ng/ml), IFN*γ* (25ng/ml), IL10 (20ng/ml) and TGF*β* (10ng/ml) (LPS from Sigma-Aldrich and all others from R&D Systems, Minneapolis, MN. CCL2 was added at the time of plating cells (0 hours), replaced with LPS at 2 hours, which was supplemented with TNF*α* at 3 hours and IFN*γ* at 7 hours. Cells were washed and placed in media containing IL10 at 14 hours, which was replaced with TGF*β* at 24 hours. The cells were kept at 37 C for the first 2 hours, shifted to 39 C until 14 hours and moved back to 37 C until the end of the experiment while being at 5% CO_2_ throughout. Cells were harvested for RNA extraction at 0, 2, 2.5, 3, 4, 7, 10, 14, 24 and 48 hours.

### RNA isolation and Quantitative RT-PCR Analysis

RNA was isolated from the samples in 96 well plates using the RNeasy 96 Kit (Qiagen) and converted into cDNA using the One-Step RT-PCR kit (Applied Biosystems). The following primers were obtained from RealTimePrimers (GAPDH forward primer: 5’-ggaaggactcatgaccacag-3’, reverse primer: 5’-ttggcaggtttttctagacg-3’; IL1A forward primer: 5’-atcagtacctcacggctgct-3’, reverse primer: 5’-tgggtatctcaggcatctcc-3’; TNIP3 forward primer: 5’-gtgcctggtcatgttttcct-3’, reverse primer: 5’-tttgtgcatccaacagcaat-3’). Transcript fold changes for IL1A and TNIP3 were calculated with respect to GAPDH.

## Supporting information

S1 Table Prediction accuracy of gene pairs in human monocytes (GSE47122) identified using *Looper*. (XLSX)

S2 Table Prediction accuracy of perturbation stage from leave-one-individual-out cross-validation (LOOCV) analysis in the Montreal cohort vaccinated with YF17D (GSE 13699). (XLSX)

S1 Fig Gene expression profiles of gene pairs tracing loops discovered in human monocytes (GSE47122). Each line represents the median gene expression across all individuals. Gene expression data is presented on a log2 scale. The gene expression profiles highlight a phase shift between the pair of genes.
(TIFF)

S2 Fig Qualitative demonstration of distinct time points in the IL1A-TNIP3 loop derived from the human monocyte dataset (GSE47122). (A) IL1A-TNIP3 loop comprised of all data points in the training and test samples. Circles represent individual samples. (B) Polar plot derived from IL1A-TNIP3 loop comprised of all data points. Points are colored based on an ordinal time scale to clearly distinguish between points sampled at different times (0 on this ordinal scale corresponds to time point 0 hrs, 1 to 2 hrs, 2 to 2.5 hrs, 3 to 3 hrs, 4 to 3.5 hrs, 5 to 4 hrs, 6 to 14 hrs, 7 to 24 hrs, and 8 to 48 hrs, respectively). Distinct time points can be seen to occupy distinct regions on the plot. (C) Angle derived from polar transformation of the IL1A-TNIP3 loop is positively correlated with time (*ρ* = 0.98).
(TIFF)

S3 Fig Randomly selected gene pairs are poor predictors of perturbation stage. Distribution of prediction accuracy for gene pairs identified as forming loops (green) versus randomly sampled gene pairs (blue). The Kolmogorov-Smirnov statistic of 0.94 and p-value of 8.75 x 10^−6^ indicates that the two gene pairs are not sampled from the same distributions.
(TIFF)

S4 Fig Experimentally validated IL1A-TNIP3 loop for monocytes of each human donor. IL1A-TNIP3 loop constructed with log2 qPCR data shown for three different donors (a-c). Each colored line represents average gene expression values across replicates at a different dose of LPS, as labeled in the index. The lines get thicker to mark progress of time.
(TIFF)

S5 Fig Topological networks in the YF-17D vaccinated Montreal cohort (GSE13699). The topological network constructed with 91 genes (0.5% of the total genes in the Montreal cohort that show the highest standard deviation) shows that the expression level of genes at day 14 returns to day 0 baseline levels. Each box reflects the network colored by number of days post-vaccination, in the order (a-e) as days 0, 3, 7, 10, and 14.
(TIFF)

S6 Fig CDC20-IFI44L loop in the YF-17D vaccinated Montreal cohort (GSE13699). Loop constructed using gene expression data (log2) in individuals in the training data consisting of 11 individuals (subject # 4, 5, 6, 7, 8, 9, 10, 20). Circles represent individuals sampled at different time points. The time point is labeled next to the appropriate circle.
(TIFF)
