# Supplementary figures and images for: Predicting position along a looping immune response trajectory
Source: PLoS One. 2018 Oct 8;13(10):e0200147. doi: 10.1371/journal.pone.0200147 (PMC6175499; doi:10.1371/journal.pone.0200147)

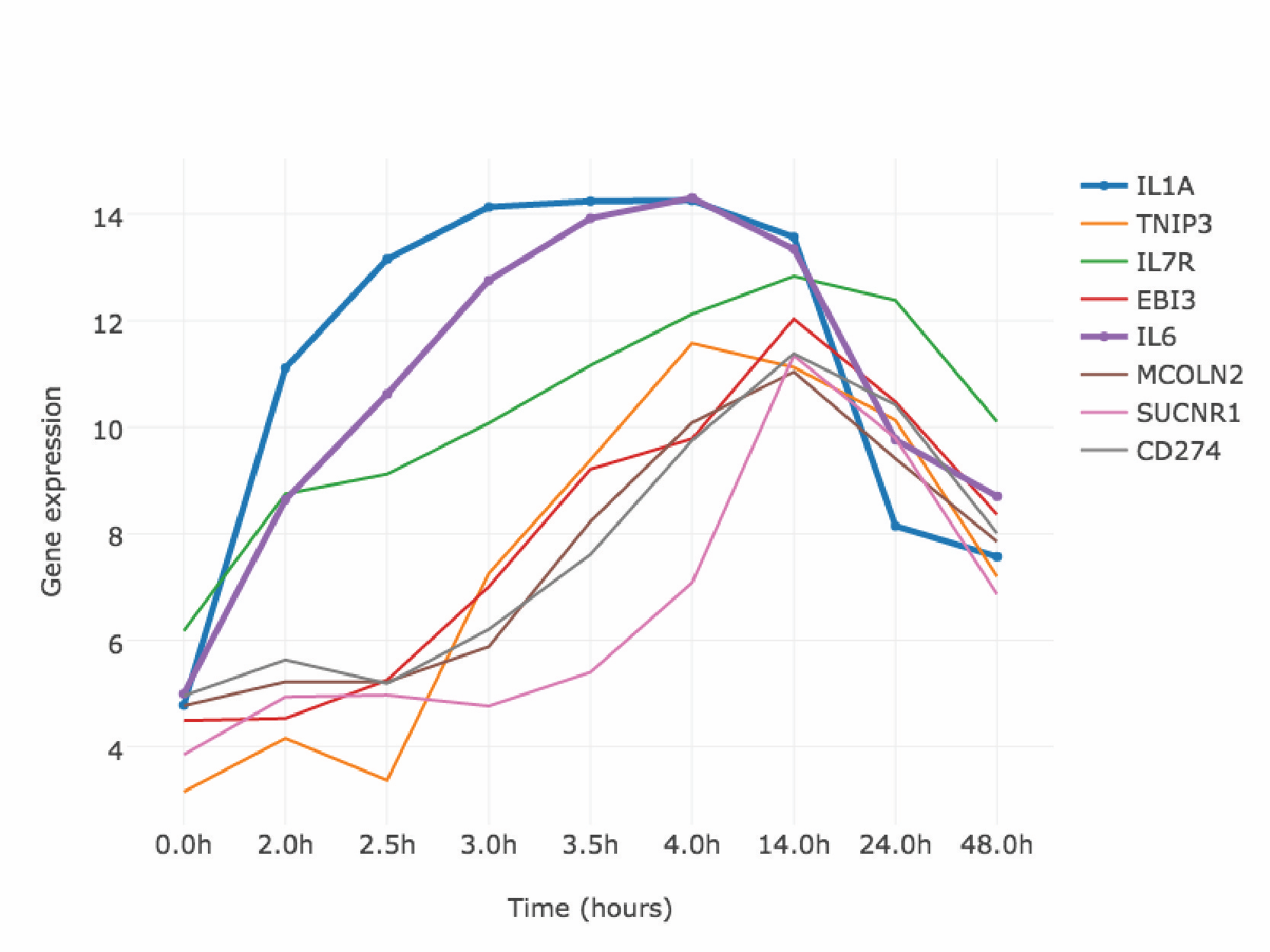

Supplement: S1 Fig — Each line represents the median gene expression across all individuals. Gene expression data is presented on a log2 scale. The gene expression profiles highlight a phase shift between the pair of genes. (TIFF) [file pone.0200147.s003.tiff]

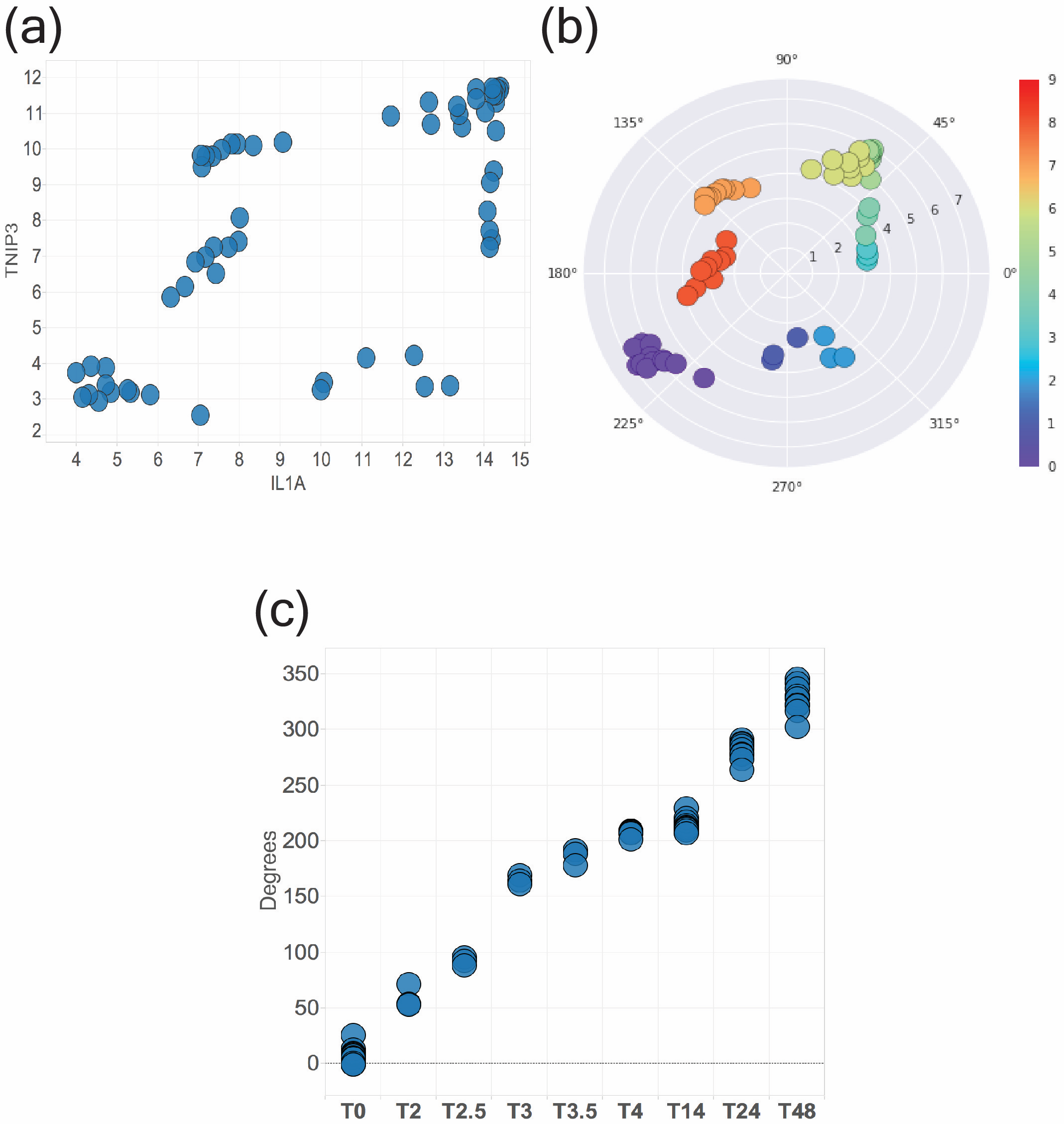

Supplement: S2 Fig — (A) IL1A-TNIP3 loop comprised of all data points in the training and test samples. Circles represent individual samples. (B) Polar plot derived from IL1A-TNIP3 loop comprised of all data points. Points are colored based on an ordinal time scale to clearly distinguish between points sampled at different times (0 on this ordinal scale corresponds to time point 0 hrs, 1 to 2 hrs, 2 to 2.5 hrs, 3 to 3 hrs, 4 to 3.5 hrs, 5 to 4 hrs, 6 to 14 hrs, 7 to 24 hrs, and 8 to 48 hrs, respectively). Distinct time points can be seen to occupy distinct regions on the plot. (C) Angle derived from polar transformation of the IL1A-TNIP3 loop is positively correlated with time (ρ = 0.98). (TIFF) [file pone.0200147.s004.tiff]

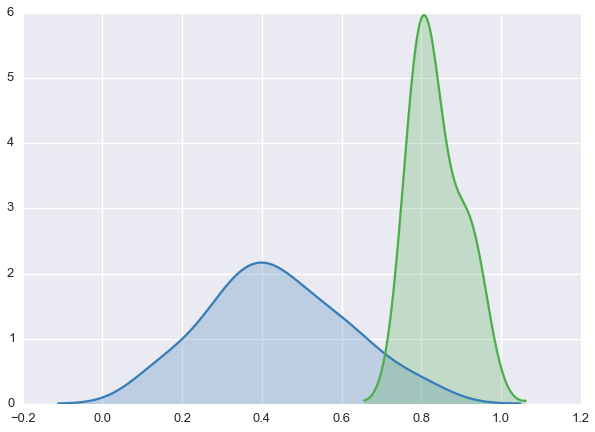

Supplement: S3 Fig — Distribution of prediction accuracy for gene pairs identified as forming loops (green) versus randomly sampled gene pairs (blue). The Kolmogorov-Smirnov statistic of 0.94 and p-value of 8.75 x 10^−6^ indicates that the two gene pairs are not sampled from the same distributions. (TIFF) [file pone.0200147.s005.tiff]

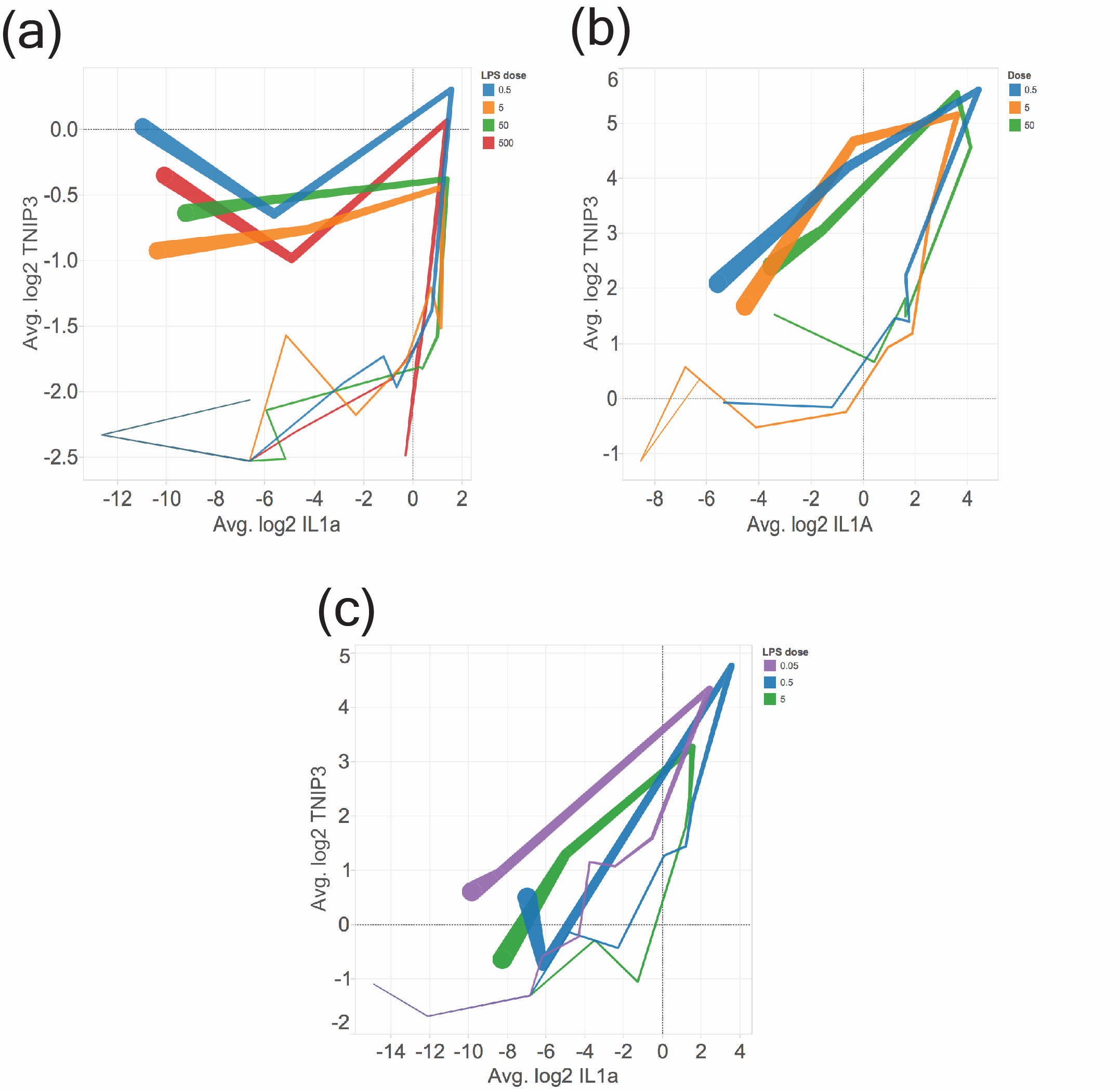

Supplement: S4 Fig — IL1A-TNIP3 loop constructed with log2 qPCR data shown for three different donors (a-c). Each colored line represents average gene expression values across replicates at a different dose of LPS, as labeled in the index. The lines get thicker to mark progress of time. (TIFF) [file pone.0200147.s006.tiff]

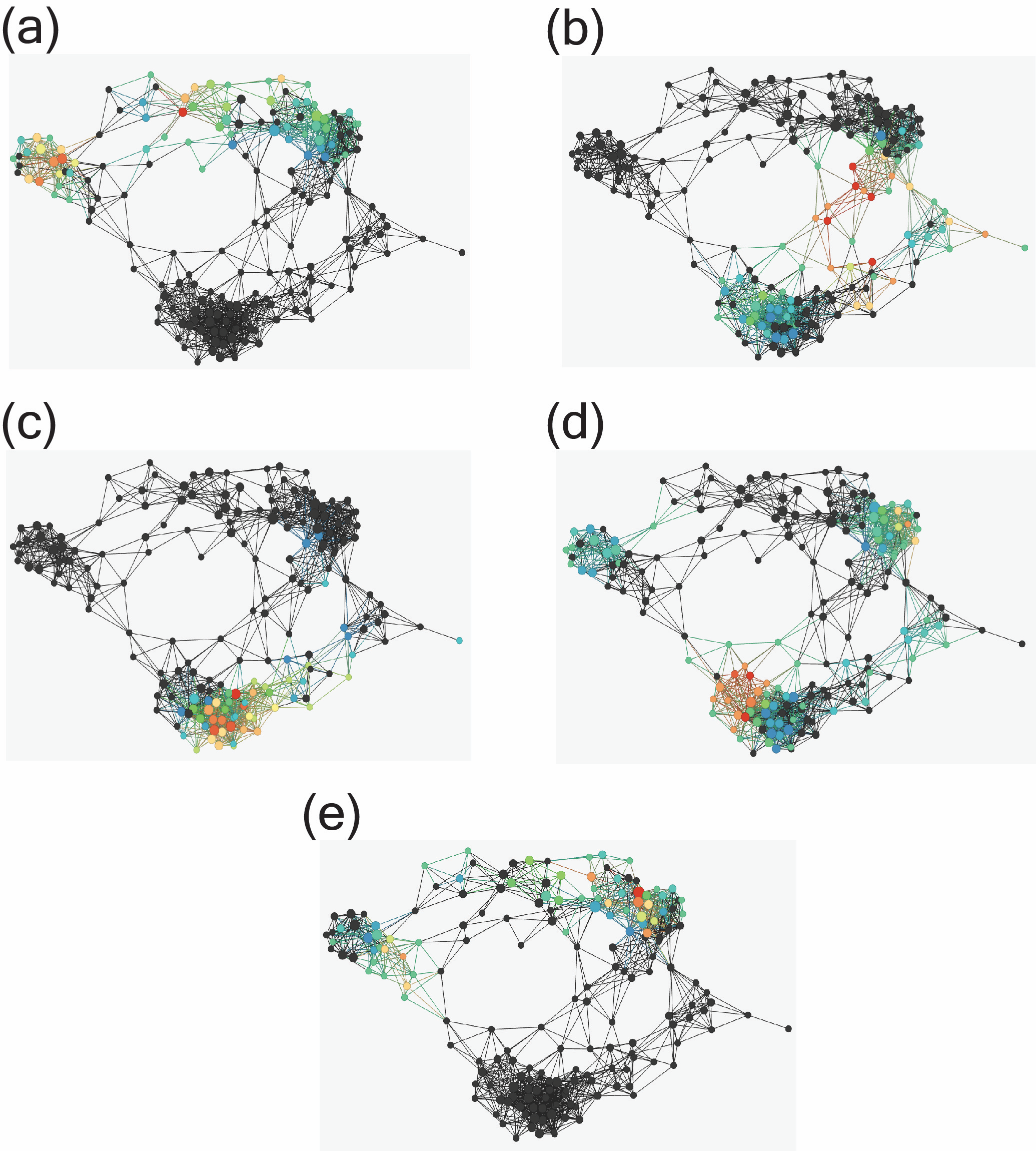

Supplement: S5 Fig — The topological network constructed with 91 genes (0.5% of the total genes in the Montreal cohort that show the highest standard deviation) shows that the expression level of genes at day 14 returns to day 0 baseline levels. Each box reflects the network colored by number of days post-vaccination, in the order (a-e) as days 0, 3, 7, 10, and 14. (TIFF) [file pone.0200147.s007.tiff]

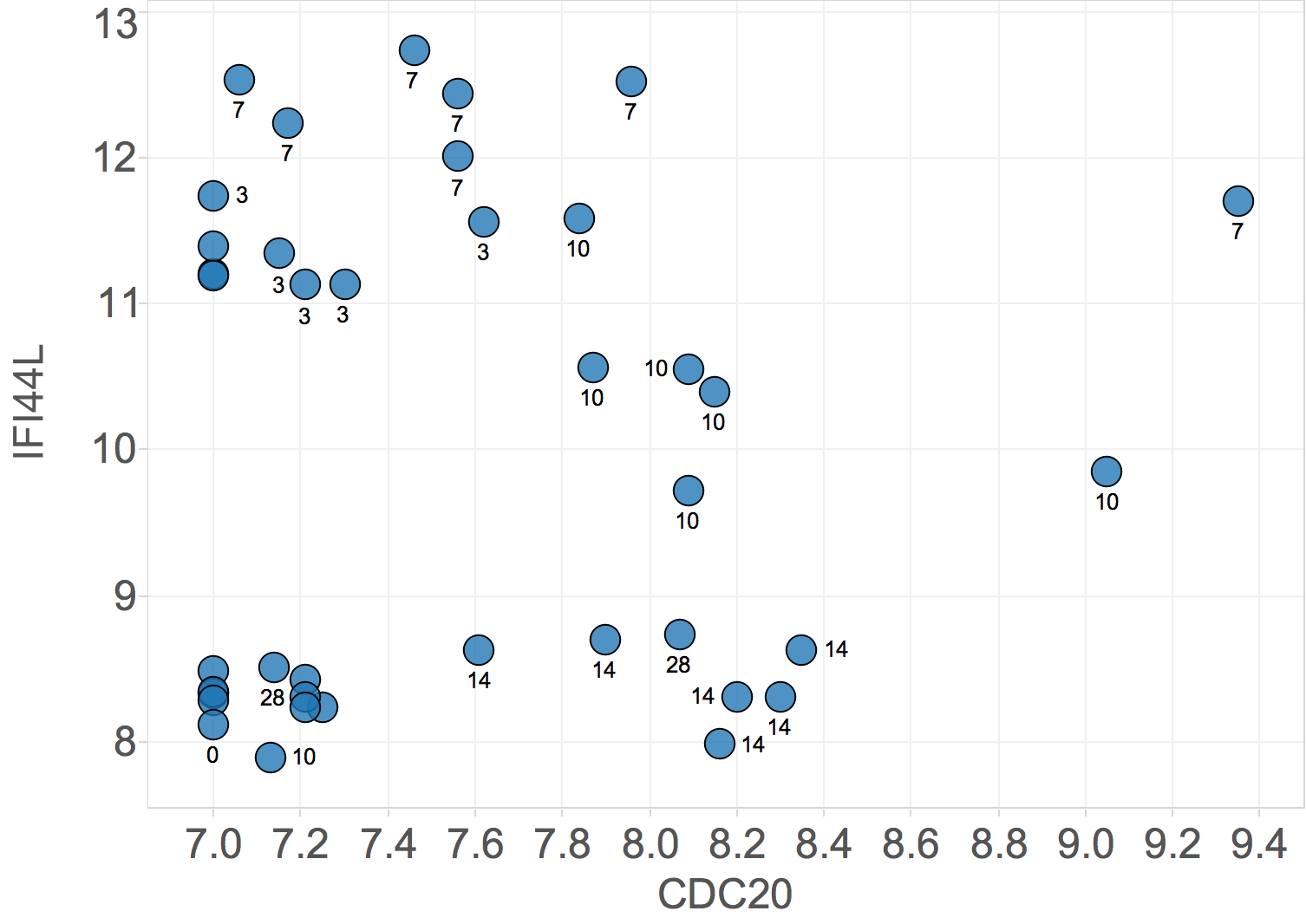

Supplement: S6 Fig — Loop constructed using gene expression data (log2) in individuals in the training data consisting of 11 individuals (subject # 4, 5, 6, 7, 8, 9, 10, 20). Circles represent individuals sampled at different time points. The time point is labeled next to the appropriate circle. (TIFF) [file pone.0200147.s008.tiff]
